# Characteristic Analysis of Unsafe Behavior by Coal Miners: Multi-Dimensional Description of the Pan-Scene Data

**DOI:** 10.3390/ijerph15081608

**Published:** 2018-07-29

**Authors:** Ruipeng Tong, Yanwei Zhang, Pengcheng Cui, Cunli Zhai, Meng Shi, Surui Xu

**Affiliations:** 1 School of Resources & Safety Engineering, China University of Mining and Technology (Beijing), Beijing 100083, China; 13121957417@163.com (Y.Z.); 18811776127@163.com (P.C.); zhaicunli@126.com (C.Z.); 2 Sustainable Minerals Institute, University of Queensland, St Lucia, Brisbane, QLD 4072, Australia; m.shi@uq.edu.au; 3 School of Safety Engineering, China University of Labor Relations, Beijing 100048, China; kerryxsr@163.com

**Keywords:** coal miner, unsafe behavior, multi-dimensional, pan-scene data

## Abstract

As a high-risk occupation, coal mining has many accidents, primarily due to the unsafe behavior of coal miners. Based on the research of analysis of unsafe behavior and pan-scenario data of miners, a theoretical framework for the analysis of unsafe behavior characteristics was proposed in this paper. The collected data were divided into realistic scenes and abstract scenes according to different manifestations; the pan-scene data were described from the eight dimensions of time, behavioral trace, location, behavioral property, behavioral individual, degree, unsafe action, and specialty using a quantitative method for the structure conversion; and the rules were discovered through cluster analysis and association analysis. A total of 225 coal mine gas explosion accidents were used for analysis, and the pan-scene data description and structure conversion of unsafe behavior that caused these accidents were realized. In a certain cluster, the distribution rules of dimensions and the interaction between different dimensions of unsafe behavior were explored after analysis. The results show that the proposed eight dimensions can fully explain the basic characteristics and attributes of the unsafe behavior of coal miners. The structure conversion can reduce the workload of managers and effectively improve the safety data processing capabilities, and the result of data analysis can provide data support and a management basis for safety management. A new method and thought for the data analysis of miners’ unsafe behavior is provided.

## 1. Introduction

In recent years, safety and health of work have gradually become the focus of attention in the development of industry [1]. Especially the coal industry, which has the most serious accidents in industrial production. Approximately one-half of Chinese major disasters occur in coal mines. The death toll from coal mine accidents exceeds that of all other accidents combined in China [2]. Coal mining is also considered one of the most dangerous jobs in the world [3]. Compared with other industries, coal mine workers face a relatively more dangerous working environment [4]. The frequent occurrence of coal mine accidents seriously affects the security situation of China’s coal industry and the sustainable development of the whole society’s economy [5].

Over the years, the Chinese government has continuously increased the intensity of safety management of coal enterprises, to a large extent, has improved the performance of coal mine safety production [6]. Many experts and scholars have also made a lot of important contributions in coal mine safety management and accident prevention. Mahdevari et al. put forward an evaluation method based on fuzzy TOPSIS to solve the health and safety problems of underground coal mine workers. This method can support the decision-making of coal mine management measures and provide an appropriate balance between different issues such as safety and cost of coal mines [7]. In the experimental study of physiological changes of personnel related to coal mine accidents, Nie et al. obtained the rules of physiological changes of injured personnel through quantitative analysis, which provided scientific basis for the training of employees’ emergency response ability in coal mine enterprises. It can effectively reduce the coal mine accidents caused by the staff’s physiological ability defect [8]. Chu et al. investigated the global problems related to coal mining, analyzed frequent accidents, occupational diseases and environmental impacts and put forward some targeted suggestions for controlling and preventing coal mine accidents [9]. Sanmiquel et al. used Weka software to analyze mining accidents in Spain and obtained some behavior patterns based on some rules, thus helping coal mining enterprises to formulate appropriate accident prevention policies and effectively reduce accidents and casualties [10]. Meanwhile, with the rapid development of the Chinese economy and the continuous construction of digital mines, the safety production techniques and the level of management of coal mines have risen. The total number of coal mine accidents and the occurrence of serious accidents have been greatly checked. However, the Chinese coal mining safety situation remains quite serious compared with the international level. The number of deaths is much higher than those in other large coal-producing countries, such as the US, Russia, South Africa, and India [11,12,13].

The reliability of humans introduces considerable uncertainty into the complex human-machine-environment system. Heinrich found that 88% of accidents were caused by unsafe human behavior from a statistical analysis of 75,000 accident cases [14]. Willamson et al. confirmed that 91% of the causes of accidents included behavioral factors after analyzing all occupational deaths from the years 1982–1984 in Australia [15]. Christina determined that the number of incidents involving unsafe behavior increased from approximately 20% to 80% after an analysis of technical system incidents from the 1960s to the 1990s [16]. Chen et al. revealed that 97.67% of coal mine accidents may be attributed to unsafe behavior [17]. Chidambaram also confirmed that investigating the human factors is of great significance to reducing the number of accidents; as a systematic and complex process, coal mining has a close relationship with people in every aspect of safe production [18]. The above rules are also applicable in coal mine accidents. More than 80% of the total number of accidents in China are caused by unsafe behavior [19]. According to the analysis of major accidents in China from 1980 to 2010, the proportion of human factors is 96.5% [20], and the unsafe behavior of human factors is the direct cause of most accidents in coal mines. Reducing the number of accidents and improving safety performances can only be achieved by systematically focusing on that unsafe behavior [21]. In the study of unsafe behavior factors in underground coal mine safety management, Paul et al. found that many unsafe factors are the direct causes of accidents and industrial injuries, and some unsafe behavior factors will still make the production of coal mine enterprises in dangerous state even if they do not lead to accidents and injuries [22]. If the unsafe behavior of miners cannot be forewarned and controlled in time, it may have serious consequences on the safety of production and safety of workers in coal mines [23]. Therefore, the study on unsafe behavior by coal miners has important practical significance for preventing and reducing the number of accidents.

Scene is the summation of the relationships between humans, surroundings and objects and can be divided into two types: a realistic scene and an abstract scene [24]. Realistic scenes include patrol photos, video data and others, while abstract scenes include accident investigation reports and related statistical data [25]. Guo et al. developed a personalized behavioral safety training system by identifying the picture scene of subway construction and established a framework system for unsafe worker behavior training [26]. Yin et al. analyzed the deep causes of the accidents by combining the behavior safety model with the reports and the cases of gas explosion accidents. As the application basis of statistical data, the specific scene includes the scene data, which can be understood as the structure statistics of the information. Based on the four dimensions of time, location, human, and action, taking the unsafe behavior of the human in the scene as the core, the internal information of the scene data is extensively explored, and the multi-dimensional description of the scene is realized to form richer pan-scene data. Wang et al. put forward the concept of emergency scene in the framework of structural error behavior analysis method in the research of subway accident investigation traffic dispatcher error behavior and put forward the structural transformation of subway accident data. It is easy for safety managers to understand the common error behavior more clearly and provide a more detailed method to collect and store the wrong behavior data in the subway traffic scheduling system [27]. Kumar et al. put forward a framework of data mining for road traffic accidents in the analysis and research of road traffic accidents. The inherent laws and hidden characteristics of road traffic accidents are analyzed by cluster analysis and other data processing methods. Based on the research of structured data, this study provided a new idea for data feature analysis [28].

The multiple accidents of a coal miner, the predominance of accidents caused by unsafe worker behavior, the complexity of the environment, and the explosion of data all propose new requirements for the traditional methods of statistical data on behavior-based safety. Improving the processing ability and analyzing the information resources to form effective management strategies and improve the level of safety management has become a topic for many scholars. Meanwhile, the theory of pan-scene data remains imperfect at home and abroad for the security sciences’ generalized data mining. There is a lack of research on the characteristics of unsafe behavior of miners based on structured data. Therefore, in this study, the unsafe behaviors of coal mine workers are taken as the research object to construct the theoretical framework for analysis of unsafe behavior characteristics based on pan-scene data. Finally, with China’s 225 coal mine gas explosion accident reports as an example, the description of the scene data and structural transformation are realized, and the regularities and the characteristics of the unsafe behavior that caused these accidents are obtained through cluster analysis and association analysis.

## 2. Theoretical Framework

**Figure 1:**
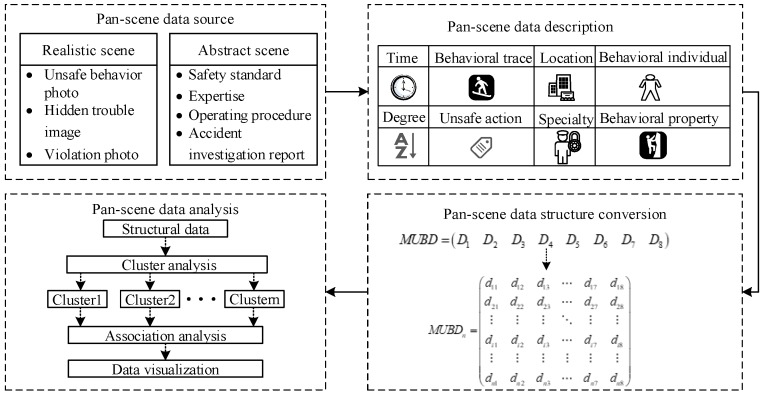
Theoretical framework for analysis of unsafe behavior characteristics based on pan-scene data.

The theoretical framework for analysis of unsafe behavior characteristics based on pan-scene data shown in Figure 1 primarily includes four parts: pan-scene data source; pan-scene data description; pan-scene data structure conversion; and pan-scene data analysis. The first part is for collecting the realistic scene and abstract scene data from a coal mine enterprise or a certain type of accident and screening the useful data resources for subsequent data processing. The second part is the description of the pan-scene data, which is done using the eight dimensions. The third part is the structural transformation of the pan-scene data. The level of concept and granularity of each dimension are clarified, and the value of each attribute is encoded and quantified. The fourth part is the pan-scene data analysis. Cluster and association analyses are used to mine data rules to realize the visualization of unsafe behavior data.

### 2.1. Pan-Scene Data Source

Scene is the purpose of statistical data application. The occurrence of unsafe coal miner behavior can be seen as a scene. According to different manifestations, pan-scene data sources can be divided into two categories:

#### 2.1.1. Realistic Scene

Behavior monitoring and observation can be used as tools for internal inspection and feedback [29]. Combined with the safety management of coal mines in China, the safety managers of coal mine enterprises will take photos to obtain evidence when they conduct safety supervisions and then fill in the inspection certificate according to the process of BBS. At the same time, coal mining enterprises in China install several camera devices for video surveillance as part of the construction of intelligent mines. Therefore, a considerable amount of unsafe behavior video data is preserved. The research value of inspection photos was gradually discovered [30]. Field inspection photos and video surveillance data can fully reflect some factors of behavior, such as place and time, which is first-hand information on the unsafe behavior pan-scene data.

#### 2.1.2. Abstract Scene

Safety standards and operating regulations are important for determining unsafe behavior [31]. Studies show an internal relationship between “rules and regulations” and behavior, and this is a finding that implies “a restriction of rules and regulations on the behavior” exists [32]. China regulates coal miner behavior by revising and improving the legal status of “coal mine safety regulations.” Coal enterprises and relevant departments have formulated “the coal mine working standard” and “the coal miners’ unsafe behavior management manual” to manage the behavior of coal miners; scholars and experts have also obtained statistics and conducted analyses on the unsafe behavior of coal miners, and they have proposed relevant management countermeasures and targeted preventive measures [33]. Furthermore, the process of the accident, the cause of the accident, the rectification measures, and the result of accident handling have been recorded in the accident investigation reports [34], which are an important source of unsafe behavior pan-scene data that includes the unsafe behavior of the human and the unsafe conditions of the material.

### 2.2. Description Model

Through the analysis of data sources of unsafe behavior, it is found that the difference between the recording mode of the realistic scene and the abstract scene and the difference between the realistic and abstract scenes in the form of unstructured or semi-structured data have made it difficult to describe unsafe behavior. Therefore, it is important to explore a method to express the coal mine unsafe behavioral pan-scene data and to standardize and quantify the data from different sources.

The unsafe coal miner behavior pan-scene data structured description should reflect the behavior itself as comprehensively as possible. Based on the Lasswell “5W analysis method,” the unsafe coal miner behavior pan-scene data description model was proposed to describe the characteristics of unsafe behavior through practice and communication with safety management workers at the coal mine site and the summary of the related literature about unsafe behavior in this paper; moreover, this method highlighted the following eight dimensions:

- Time: describe the time when the unsafety behavior occur.
- Behavioral trace: describe the traceability of unsafety behavior.
- Location: describe the location of the area where the unsafety behavior occur.
- Property: describe the type of unsafety behavior.
- Behavioral individual: describe the individual who have an unsafety behavior.
- Degree: describe the severity of unsafety behavior.
- Unsafety action: describe the specific unsafety behavior.
- Specialty: describe the stage where the unsafety behavior occurs and the specialty of the coal mine work operation.

### 2.3. Structure Conversion

The description of unsafe behavior in coal mines is more systematic and standardized through the model. However, it does not achieve a structured and coded quantitative description. Therefore, it is necessary to analyze the level of concept and the value of attributes for each dimension of the unsafe behavior guided by the method of quantification.

#### 2.3.1. Time (T)

The continuity of time is a common characteristic in the development of anything. Some scholars have extensively studied the rules of time for human behavior, and these rules are used in various fields [35,36]. It is also important for safety management to define the rules for the occurrence time of unsafe behavior through pan-scene data mining. The time dimension has a different division for granularity and levels, such as quarter, month, day, and shift. The smaller the granularity is, the lower the level. Predictions from low levels to high levels can be achieved to improve the accuracy of information based on statistical data. Based on the characteristics of the information expressed by the source data, the structural conversion of the time dimension is shown in Table 1 combined with the reality of the coal mine.

#### 2.3.2. Behavioral Trace (BT) 

Behavioral trace indicates the traceability after the occurrence of the behavior. Based on the behavioral trace, the behavior can be divided into traced unsafe behavior and non-traced unsafe behavior. The characteristic of traced unsafe behavior is that the certain behavioral traces will be left after the occurrence of the behavior within a certain period of time; in contrast, the characteristic of non-traced unsafe behavior is that the unsafe behavior can only be found as it occurs and will not be traceable. Safety managers can use appropriate methods to infer the cause of unsafe behavior according to the characteristics of different behavioral traces. For the traced unsafe behaviors, the focus is on the identification of responsibility and the corresponding timely punishment. However, for the non-traced unsafe behaviors, the focus is to strengthen the supervision and inspection of the field. Therefore, it is particularly necessary to take behavioral traces as a dimension of unsafe behavioral pan-scene data. The structural conversion of the behavior trace dimension is relatively simple: Bt1 represents traced unsafe behavior, and Bt2 represents non-traced unsafe behavior.

#### 2.3.3. Location (L)

This dimension is used to identify the location of the region where the unsafe behavior occurs. Relevant studies have shown that work environments have a significant effect on the behavioral choices of workers [37,38]. Therefore, the study on the dimensions of work sites is of great significance. Based on the characteristics of accidents in the past and the location where unsafe behavior in coal mines occurs frequently, similar to the time dimension, the regional locations are stratified according to different levels of granularity to clarify the differences in the occurrence of unsafe behavior and to improve safety management efficiency. Combined with the reality of the coal mine, the structural conversion of the location dimension is shown in Table 2.

The names of the accident sites in this research are standard, for they are derived from Terms relating to coal mining (GB/T 15663-2008), National Standard of the People’s Republic of China.

#### 2.3.4. Behavioral Property (BP) 

Behavioral property reflects the category of unsafe coal miner behavior combined with relevant research [39,40], including violation of commands, violation of operation, violation of action and non-violation unsafe behavior. Among them, violation of commands refers to the behavior of management personnel who order other people to conduct illegal operations; violation of operation refers to the behavior of the workers who violate the operating procedures to operate the specific object, such as “the sledgehammer of dangerous rock”; violation of action refers to the unsafe actions of the workers who do not involve the object, equipment or facility in the work, such as “the distance from the blasting does not meet the requirement”; and non-violation unsafe behavior refers to behavior that does not violate laws and regulations (excluding the company’s internal regulations), but the action itself is unsafe and provides impetus to the occurrence of accidents, such as “without careful inspection after blasting.” The structural conversion of the behavioral property is as follows: Bp1 represents violation of command, BP2 represents violation of operation, BP3 represents violation of action, and Bp4 represents non-violation unsafe behavior.

#### 2.3.5. Behavioral Individual (BI) 

The behavioral individual dimension refers to the different individual attributes of the worker’s unsafe behavior. Factors such as age [41,42], working age, and physical status at work all affect unsafe behavior [22,43]. This paper only analyzes the three attributes of age, working years, and job type in the individual dimension. According to the division of the property of unsafe behavior and the function, behavioral individuals can be divided into three categories: managers, field commanders, and grassroots workers. The structural conversion of the behavioral individual is shown in Table 3.

Age and working years can be queried in the coal company employee management system. However, the classification of coal mines for job type remains unclear. Through the investigation of key coal mines in major coal-producing regions in the provinces of Shanxi, Anhui, Inner Mongolia Autonomous Region, and Henan in China, the classification of job types is shown in Table 4 combined with the “Management Regulations for the Examination and Evaluation of Safety Technologies for Special Operators” promulgated by the “State Administration of Work Safety” on 26 April 2010 in China. Thus, the grassroots workers are coded according to job type in Table 4.

#### 2.3.6. Degree (D)

The degree dimension is used to reflect the severity of unsafe behavior. Based on the more mature division method of accident classification and hidden risk classification, and the analysis and assessment of the risk [44], the definition of degree dimension is the level of risk combining the practice of coal mine safety management, which is based on the direct or indirect potential severity of the consequences of unsafe behavior. Thus, the unsafe behavior is divided into five levels: serious-risk, major-risk, medium-risk, general-risk, and low-risk levels. The structural conversion is shown in Table 5.

#### 2.3.7. Unsafe action (UA) 

The unsafe action dimension describes the specific unsafe behavior that may lead to accidents, casualties and environmental disruption. According to the purpose of the action, the unsafe actions are divided into four categories: safety, operation, management, and general type. The structural conversion of unsafe action is shown in Table 6.

#### 2.3.8. Specialty (S)

The specialty dimension is used to represent the work stage and the specialized category of the coal miner when unsafe behavior occurs. According to the “Coal Mine Safety Risk Pre-control Management System” promulgated by the “State Administration of Work Safety” in 2011, the coal mine production system is divided into 14 management elements. Combining the characteristics of the unsafe coal miner behavior, causes of accidents, and previous research results [45], the specialty dimensions are divided into heading, mining, electric, transportation, “one ventilation and three preventions,” waterproofing, blasting, and others. The structural conversion of the specialty is shown in Table 7.

### 2.4. Analysis and Visualization

The pan-scene data analysis focuses on the study of the rules of distribution for unsafe coal miner behavior and the interaction between the dimensions to realize the explicitness of the interaction relationship, which can not only deeply explore the miners’ unsafe behavior in the specific coal mine enterprise but also lay a foundation for the discovery of the inherent nature of the unsafe behavior.

Before mining accident data, it is necessary to eliminate the heterogeneous nature of the data [46]. Cluster analysis is used to preliminarily explore the distribution of accidents and to prepare for multi-dimensional correlation analysis. The use of multi-dimensional interaction analysis in different clusters can deeply explore the interaction relationship between different dimensions, which is of great significance for discovering the potential characteristics of unsafe behavior.

According to the eight dimensions of the unsafe coal miner behavior pan-scene data, theoretically, any two or more dimensions can be analyzed. The interaction between different dimensions has different practical significance, which can explore the deep rules of unsafe behavior and improve safety management efficiency. Some representative dimensions for association analysis and the meaning of safety management are shown in Table 8.

## 3. Materials and Methods

### 3.1. Data and Structure Conversion

The accident investigation reports were used for analysis. This paper selects 225 cases of gas explosion accidents in China [47]. To ensure the accuracy of the data, this paper follows the principles of accident completeness and case authoritativeness: The completeness refers to the complete content of the accident investigation report and to facts that are expressed clearly to analyze the human factors according to the report content. Furthermore, authoritativeness refers to the accident report that must be issued by the state-accredited accident investigation or safety regulatory institutions.

Because the description of unsafe behavior in the original accident investigation report is in unstructured text records, it must be adjusted to the coal mine unsafe behavioral pan-scene data description model to achieve the structural conversion. Three coal mine gas explosion accidents were selected as an example, and the results of the description and structural conversion processes are shown in Table 9.

At this point, the coal miners’ unsafe behavior pan-scene data (MUBD) based on the gas accident investigation report was constructed to include a total of 871 data points as shown in Formula 1. The pan-scene data can enable coal mining companies to record the unsafe behavior of workers more accurately and provide a method for evaluating the record for unstructured or semi-structured data on unsafe behavior and a way to analyze and visualize it.

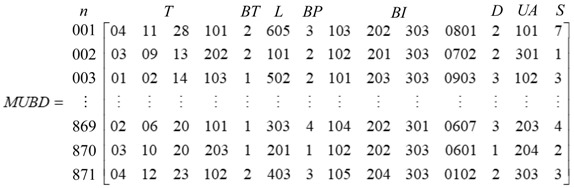
(1)

### 3.2. Clustering Method

Data mining is a fast and effective way to find unknown, implicit and potentially useful information from large-scale data to guide decision making. It is necessary to select a suitable tool to mine useful information and knowledge with strong application value from a large amount of unsafe behavior data of miners. As an unsupervised learning method, clustering plays an important role in the data natural grouping structure and has been widely used. In the efficiency of the algorithm and clustering effect, the existing cluster methods have achieved excellent performance. The k-means algorithm has been widely used for many years because of its high efficiency in data processing and good clustering effect in numerical data. However, the k-means algorithm can only evaluate a data set with continuous attributes, and the structured data presented here represent a discrete type dataset that contains many classification attributes. Thus, the k-modes algorithm is selected, which realizes the high-efficiency of the k-means algorithm while realizing the clustering of the discrete-type data [48].

In the k-modes algorithm, the difference factor is used to replace the distance in the k-means algorithm, and the smaller the difference factor is, the closer the distance will be. The difference factor between a sample and a cluster center is the number of different attributes: different values are represented by 1, identical values are represented by 0, and finally, the sum of the different values is calculated. The resulting value is the difference factor between a sample and the corresponding cluster center.

Given a data set *Z*, each data point is described by n classification variables, and the difference factor between *X* and *Y* is calculated as follows:

(2) $$d(X,Y)=\sum_{i=1}^{n}\delta (X_{i},Y_{i})$$

(3) $$\delta (X_{i},Y_{i})=\left\{ \begin{array}{l} 0,\;X_{i}=Y_{i}\\ 1,\;X_{i}\neq Y_{i} \end{array}\right.$$

where *X_i_* and *Y_i_* are the values of attribute *i* in *X* and *Y*, respectively.

The k-modes process is as follows:

Step 1. Select k objects as the cluster center.
Step 2. Use Equation (1) to calculate the difference value of each sample and cluster center.
Step 3. Distribute the samples to the cluster with the lowest difference factor between the cluster center and the cluster center. After all samples are divided, the cluster center is redetermined.
Step 4. Repeat steps 2 and 3 until the sum of the distance between each cluster sample and each cluster center is no longer reduced and retain the final cluster results.

### 3.3. Association Rules Method

Association rules are the techniques applied to large-scale database mining. The core of the association rules is to show the rules of association and the correlations between different items, which is an implication of the form: *A*⇒*B*. *A*⇒*B* represents the association between *A* and *B*: *A* is the left-hand side (*LHS*); B is the right-hand side (*RHS*). Support, confidence, and lift are three important parameters in the association rules. *I* = {I_1_, I_2_, …, I_m_} is a set including m different items, and *I* is the itemset.

Support is the probability that the elements included in the *LHS* and *RHS* are simultaneously present, that is, the ratio of the number of occurrences of itemsets *A* and *B* in the database (*A*∩*B*) to the total itemset, which can be expressed as:

(4) support(A⇒B)=count(A∩B)/count(I)

The support reflects the strength of the association rules. The minimum support is the minimum support threshold of the itemset, which is denoted *SUPmin* and represents the minimum importance of the association rules.

Confidence is the probability that *B* is extrapolated by the association rule *A*⇒*B* under the condition of containing *A*, which can be expressed as:

(5) confidence(A⇒B)=support(A∩B)/support(A)

The association rules with high support and confidence can more clearly illustrate the problem in general. The minimum confidence is denoted *CONFmin*. When the association rule *A*⇒*B* satisfies the support ≥*SUPmin* and the confidence ≥*CONFmin*, the *A*⇒*B* is called the strong association rule. The purpose of association rule mining is to find strong association rules and provide decision making assistance.

The lift reflects the size of the *RHS* influenced by the *LHS*. When the lift exceeds 1, it means that the *LHS* has a great influence on the *RHS*, and this association rule has obvious practical significance; in contrast, when the lift is less than 1, it means that the probability of *RHS* under the condition of *LHS* influence is smaller than the prior probability, and this association rule has no meaning in reality. Moreover, when the lift equals 1, it means that the leader and the successor are independent, and there is no relationship between them. The lift is the ratio of the confidence of *A*⇒*B* to the support of B, which can be expressed as:

(6) lift(A⇒B)=confidence(A⇒B)/support(B)

In this paper, we use the Apriori algorithm proposed by Agrawal to mine mining association rules from the clustering data set for the unsafe behavior of miners [49].

## 4. Results and Analysis

### 4.1. Cluster Results

The primary problem of clustering analysis is determining the number of clusters formed by clustering algorithms. In this paper, Akaike information criteria (AIC), Bayesian information criteria (BIC) and consistent AIC (CAIC) are used to judge [28]. After trying to divide several models, the data clustering number was determined to be 4. After determining the number of clusters, the k-modes algorithm was used to classify the data sets using R software. Then, each of the separated clusters was characterized. Brief descriptions of the clusters and their sizes are shown in Table 10.

#### 4.1.1. Cluster 1

Such unsafe behaviors are primarily in the tunneling working site and coal face, including 78% of the high-risk behavior; additionally, approximately 53% are traced unsafe behaviors, and most of the time is distributed in March, April, November, and December, and the operation class accounted for 77%.

#### 4.1.2. Cluster 2

Such unsafe behaviors in the airway and main haulage roadway account for 69%, and safety inspection work accounts for 82%. Additionally, 66% of behaviors occur in November and December, and most of the work types are gas inspectors and mine ventilators.

#### 4.1.3. Cluster 3

Approximately 70% of these unsafe behaviors occurred in the skip loading pocket and underground locomotive repair room, and they involved electric specialty unsafe behavior, accounting for 56%. Additionally, the working age in 0–5 years accounted for 68%.

#### 4.1.4. Cluster 4

Among these unsafe behaviors, managers and field commanders accounted for 71%, low-risk unsafe behavior accounted for 60%, general-risk unsafe behavior accounted for 58%, and the distributions of region and time were relatively wide.

The k-modes clustering method was used to analyze the unsafe behavior data of 871 miners. After detailed analysis of all the classification results, it was found that the eight dimensions describing the unsafe behavior of miners could be effectively used as the main variables of the data gathering class, and there was a significant correlation among the dimensions of the unsafe behavior of the miners.

### 4.2. Association Results

**Figure 2:**
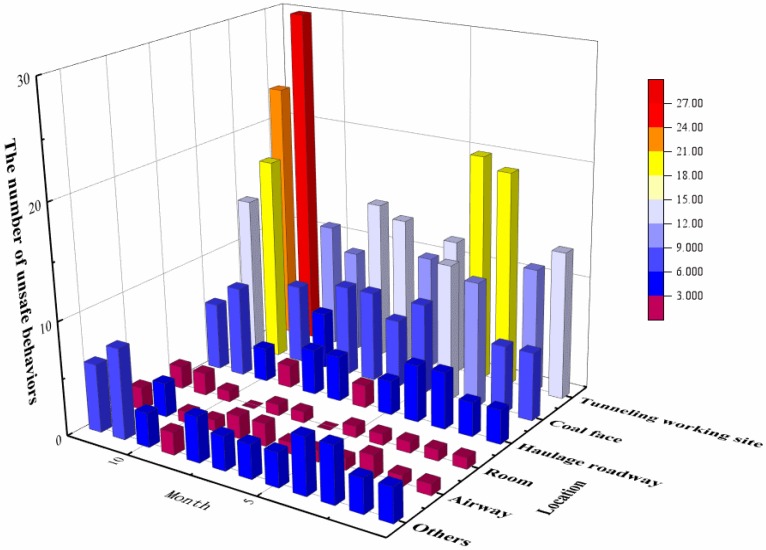
Two-dimensional analysis of time and location using data from the gas explosion accident investigation report.

Selecting the data set of Cluster 1 for association analysis and taking the time and location dimensional association analysis as an example, the statistics for the number of unsafe behaviors at different locations in different months are shown in Figure 2. It is seen from the figure that the number of unsafe behaviors in the tunneling working site and coal face was highest in November. Additionally, the number of unsafe behaviors in the tunneling working site in March, April, and December was significantly larger than the number that occurred in other locations. Moreover, for the room and airway, the number of unsafe behaviors per month was relatively small compared to the other locations. Therefore, when safety managers conduct behavior interventions, such as behavioral observations, they can determine from this figure the intensity and proportion of the number of unsafe behaviors in different locations at different times.

IBM SPSS Modeler 18.0 software (International Business Machines Corporation, Armonk, NY, USA) was selected as an association rule mining tool. Through the Apriori algorithm, the unsafe action dimension is used as the *LHS*, and the other dimensions are the *RHS*. The most prevalent unsafe behaviors in different dimensions are explored. *SUPmin* and *CONFmin* were 8% and 20%, respectively, and this information was used to obtain effective strong association rules. The results are shown in Table 11.

Combining the meaning of the attributes in each dimension, the results of the association analysis of the data are analyzed.

#### 4.2.1. Rule 1

This rule indicates that unsafe behavior occurring in November usually includes casually turning the auxiliary ventilating fan on and off. The confidence rate of this rule is 23.92%. According to this rule, the safety management efficiency can be improved 1.87-fold by behavioral intervention on workers who operate auxiliary ventilating fans casually.

#### 4.2.2. Rule 2

This rule indicates that traced unsafe behavior of gas explosion accidents usually include not evacuating in the case of reduced or stopped air. The confidence rate of this rule is 35.67%. According to this rule, the efficiency of inspection of this unsafe behavior can be improved 1.76-fold.

#### 4.2.3. Rule 3

This rule indicates that unsafe behavior at the coal face usually involves not examining the gas concentration at shift time. The confidence rate of this rule is 43.71%. According to this rule, the efficiency of controlling unsafe behavior can be improved 2.70-fold.

#### 4.2.4. Rule 4

This rule indicates that the most vulnerable unsafe behavior of gas inspectors is blasting without examining the gas concentration. The confidence rate of the rule is 52.34%. According to this rule, the efficiency of targeted safety training for gas inspectors can be improved 2.15-fold.

#### 4.2.5. Rule 5

This rule indicates that when there is a major risk of unsafe behavior, it usually involves blasting without examining the gas concentration. The confidence rate of this rule is 26.86%. According to this rule, the efficiency of controlling unsafe behavior that is of major-risk can be improved 1.89-fold.

#### 4.2.6. Rule 6

This rule indicates that when unsafe behavior occurs during the heading process, it usually involves unqualified hole-sealing. The confidence rate of this rule is 44.73%. According to this rule, the efficiency of the behavior observation of the tunneling working site can be improved 1.56-fold.

## 5. Discussion

In this paper, the investigation reports of coal mine gas explosion accidents are taken as the data source. Through the theoretical framework of unsafe behavior characteristic analysis based on the pan-scene data, the eight dimensions of time, behavioral trace, location, behavioral property, behavioral individual, degree, unsafe action and specialty are used to describe unsafe behavior, and the data structure conversion is realized. Through cluster analysis and association analysis, the unsafe behavior characteristics of coal miners are obtained and the guiding significance of the results for coal mine safety management are analyzed. Coal mining remains a high-risk occupation [50], and coal mine safety data have complex, dynamic and heterogeneous, fuzzy and random characteristics [51]. Meanwhile, digital and intelligent mine construction propose new requirements for safety management work. Based on this situation, the findings in this paper have significant implications for safety research.

The process of describing miner unsafe behavior and constructing structured pan-scene data are conducive to promoting coal enterprises to collect unsafe behavior data and manage safety information resources. In addition, this information can be used to explore the value of existing resources of behavior-based safety.

The application of new technologies and the increased emphasis on safety by the government has resulted in a decrease in the fatality rate of coal mines in China [52,53]. The construction of digital and intelligent mines proposes new requirements for coal mine safety management. The intelligent safety management must pay more attention to the application and mining of data resources. The massive data sources of unsafe behavior contain a wealth of hidden knowledge of rules and laws. However, due to the lack of extraction of coal mine unsafe behavior data, these knowledge treasures cannot be used to guide safety management. However, the analysis and mining of data primarily focuses on the construction of a data mining model and algorithm and the application of computer technology [54,55]. For the large safety production dataset, these methods are relatively weak, which restricts the extraction of safety data and the improvement of safety management. In response to this type of problem, the description of pan-scene data and the process of data structure conversion in the framework greatly improve the processing capabilities for large datasets and is of great significance to the effective use of information resources and the intelligent management of a coal mine.

For coal mine safety managers, the structured processing of behavior safety data eases the burden on managers in terms of collating photos and records of unsafe behavior. In addition, the statistical analysis and multi-dimensional association analysis results help safety managers to clarify the characteristics and rules of unsafe behavior of workers, which improves the efficiency of safety management and optimizes the allocation of management resources.

The occurrence of unsafe coal miner behavior has intrinsic complexity and is greatly affected by the natural environment, coal mining technology, personality traits, and management level [22]. Using clustering analysis, the potential rules among the dimensions of unsafe miner behavior can be preliminarily excavated, and the heterogeneity of much unsafe miner behavior data can be effectively eliminated at the same time. It is of great significance to determine the potential features of unsafe behaviors using association analysis to explore the relationship between the different dimensions in a particular single cluster. The interaction between different dimensions has different meanings. Safety managers can select the different dimensions according to their needs and judge whether there is regularity between them through the interaction results to improve the efficiency of safety management and stimulate the advancement of empirical safety management to intelligent safety management.

For the workers of coal mining enterprises, the visual presentation of the data analysis results has improved their level of information cognition. The results of cluster analysis and association analysis of eight dimensions are mostly displayed in visualization, which has characteristics that can be summarized as visibility, interactivity, and multidimensionality. Based on the perspective of safety cognition and the different individual attributes, the results will be provided to the workers to improve their information absorption and analysis ability and their safety behavior selection ability.

## 6. Conclusions

The theoretical framework for analysis of unsafe behavior characteristics based on pan-scene data in this study has very important application value. The requirement of the pan-scene data theory to the accident data source can maximize the comprehensive description of human unsafe behavior factors. The accuracy and completeness of unsafe behavior data are guaranteed. Thus, the unsafe behavior information associated with the accident can be extracted more thoroughly. The data structure conversion process can be used not only to follow-up analysis but also to improve the management and storage of accident data in coal mining enterprises.

Through cluster analysis and correlation analysis, the safety managers of coal mining enterprises can directly understand the probability distribution of all kinds of accidents and the interrelations and interaction rules between the various dimensions of the unsafe behavior of the workers. In practical work, the analysis results of unsafe behavior characteristics can provide a scientific basis for the rational allocation of safety management and accident prevention resources and the adjustment of safety training programs in coal mining enterprises. These targeted guidance functions are conducive to greatly improving the safety performance of coal mining enterprises.

Based on the study of unsafe behavior of miners [19,20], this paper realizes the structure conversion of data based on the theory of pan-scene data [24,25,26] and introduces the framework thought of data characteristic analysis into the field of coal mine [27,28]. A framework for analyzing unsafe behavior characteristics of workers in coal mines is formed, and the feasibility and effectiveness of the framework are verified by accident cases. In this paper, there is a solid theoretical foundation and a sufficient empirical analysis, the description of data sources is more comprehensive, and the design of structure conversion and characteristic analysis process are more suitable for coal mines. A new method and thought for the data analysis of workers’ unsafe behavior is provided in coal mines.

This paper hopes to contribute to the characteristics analysis of unsafe behaviors of coal mining enterprises by proposing a data processing and analysis method. However, there are limitations in this study. The data sample selected in this paper is only the coal mine gas explosion accident from 2001 to 2016 in China. There are various types of coal mine accidents with different characteristics. Therefore, the results of this study have certain limitations, which will become the focus of our subsequent research.

## Figures and Tables

**Table 1:** Structured conversion of the time dimension of unsafe coal miner behavior pan-scene data.

Coding	Time	Value
T1	Quarter	01 = First quarter; 02 = Second quarter; 03 = Third Quarter; 04 = Fourth quarter
T2	Month	01 = January; 02 = February; 03 = March; 04 = April;…; 12 = December
T3	Day	01 = 1; 02 = 2; 03 = 3; …; 31 = 31
T4	Shift	“three and eight” working form: 101 = morning shift; 102 = night shift; 103 = maintenance shift
		“four and six” working form: 201 = morning shift; 202 = afternoon shift; 203 = night shift; 204 = maintenance shift

Note: “three and eight” working form: three shifts including two working shifts and one maintenance shift, which work eight hours per shift every day; “four and six” working form: four shifts including three working shifts and one maintenance shift, which work six hours per shift every day.

**Table 2:** Structured conversion of the location dimension of coal miner unsafe behavior pan-scene data.

Coding	Location	Value
L1	Tunneling working site	01 = Tunneling working site roadway; 02 = Tunneling working face
L2	Coal face	01 = Working face; 02 = Head gate; 03 = Tailgate; 04 = Goaf
L3	Haulage roadway	01 = Main haulage roadway; 02 = Haulage drift; 03 = Centralized main roadway; 04 = Main roadway for single seam
L4	Room	01 = Skip loading pocket; 02 = Underground locomotive repair room; 03 = Shaft coal pocket; 04 = Main pumping room; 05 = Underground charging station
L5	Airway	01= Main return airway; 02 = District return airway; 03 = Section return airway; 04 = Ventilation shaft
L6	Others	01 = Crossheading; 02 = Cross-cut; 03 = Shaft bottom; 04 = Open-off cut; 05 = Blind roadway; 06 = Others

Note: (1) Divide the tunneling working site into two parts: tunneling working site roadway and tunneling working face. Tunneling working face refers to the range of the region where the heading is continuing but the roadway is not permanently supported; the tunneling working site roadway refers to the roadway at the tunneling working site except the tunneling working face. (2) This paper defines the coal face as a space for direct coal work and mining including four parts: working face, head gate, tailgate, and goaf.

**Table 3:** Structured conversion of the behavioral individual dimension of the unsafe coal miner behavior pan-scene data.

Coding	Behavioral Individual	Value
BI1	Age (year-old)	01 = 20–30; 02 = 30–35; 03 = 35–40; 04 = 40–45; 05 = 45–50; 06 = Over 50
BI2	Working age (years)	01 = 0–5; 02 = 5–10; 03 = 10–15; 04 = 15–20; 05 = 20–25; 06 = Over 25
BI3	Function	01 = Managers; 02 = Field commanders; 03 = Grassroots workers

**Table 4:** Classification and structural conversion of grassroots workers in a coal mine.

Coding	Type	Value
01	Transportation	01 = Mine track worker; 02 = Hauler; 03 = Conveyor operator; 04 = Hoist driver; 05 = Mine winding operator;
02	Geologic measurement	01 = Driller; 02 = Mine geologic worker; 03 = Mine measurement worker
03	Outburst prevention	01 = Gas outburst prevention worker; 02 = Water injection worker
04	Gas extraction	01 = Gas extraction worker; 02 = Drilling machine operator; 03 = Gas pump operator
05	Blasting	01 = Blaster; 02 = Mine powder magazine worker
06	Mining	01 = Coal mining worker; 02 = Support worker; 03 = Coal winning machinery driver; 04 = Crusher driver; 05 = Hydraulic support worker; 06 = Roadway repairman; 07 = Common worker
07	Safety check	01 = Safe inspector; 02 = Ventilator; 03 = Roadway excavation and masonry worker; 04 = Explosion-proof electric apparatus inspector; 05 = Bolting and shotcreting worker
08	Gas inspection	01 = Gas inspector; 02 = Mine dust testing worker; 03 = Mine dust removal worker
09	Electric	01 = Electric installer; 02 = Electric operator; 03 = Electric maintenance worker
10	Safety observation and monitoring	01 = Observation and monitor operator; 02 = Safety instrument monitor; 03 = Equipment maintenance worker

**Table 5:** Structured conversion of degree individual dimension of unsafe coal miner behavior pan-scene data.

Coding	Degree	Explanation
D1	Serious risk	Unsafe behavior that is highly likely to cause serious accidents and is extremely difficult to repair afterwards
D2	Major risk	Unsafe behavior that may cause serious accidents and is difficult to repair afterwards
D3	Medium risk	Unsafe behavior that may cause major accidents and is difficult to repair afterwards
D4	General risk	Unsafe behavior that may cause general accidents and is not difficult to repair afterwards
D5	Low risk	Unsafe behavior that is less likely to cause accidents and is easy to repair afterwards

**Table 6:** Structured conversion of the unsafe action dimension of unsafe coal miner behavior pan-scene data.

Coding	Unsafe Action	Value
UA1	Safety	01 = Safety inspection; 02 = Safety working; 03 = Hidden trouble treatment; 04 = Instruction; 05 = Escape; 06 = Adventure
UA2	Operation	01 = Supporting; 02 = Heading; 03 = Blasting; 04 = Mining
UA3	Management	01 = Regulatory measures; 02 = Labor organization; 03 = Monitoring
UA4	General type	01 = General-type

**Table 7:** Structured conversion of the specialty dimension of unsafe coal miner behavior pan-scene data.

Coding	Specialty	Explanation
S1	Heading	The unsafe behavior occurs during operations such as heading, roof supporting, and refuse transportation at the tunneling working site
S2	Mining	The unsafe behavior occurs during operations such as coal mining, supporting, and transportation primarily at the coal face
S3	Electric	The unsafe behavior occurs during the operation, maintenance, and inspection of machinery and equipment
S4	Transportation	The unsafe behavior occurs during the transportation of coal or refuse at the main transportation roadway
S5	One ventilation and three preventions	The unsafe behavior occurs during the ventilation, dust prevention, fire prevention, and gas prevention
S6	Waterproof	The unsafe behavior occurs during operations such as water inspection, water exploration, water discharge, water blocking, and water interception
S7	Blasting	The unsafe behavior occurs during the blasting, including materials, equipment, and operations
S8	Others	In addition to the above seven types of unsafe behavior

**Table 8:** An example of unsafe coal miner behavior pan-scene data association analysis.

Interaction Dimension	Explanation	Practical Significance
T ↔ L	Are there any regularities in the locations where unsafe behavior occurs at different times?	Determine the time period when unsafe behavior in different regions is likely to occur in different locations, and then strengthen inspections.
BI ↔ S	Are there any regularities in the stages and professions of different individuals’ unsafe behavior?	Identify which individuals in different stages are prone to unsafe behavior, and then strengthen the intervention at a certain stage.
BP ↔ D	Are there any differences in risk level between different behavioral properties?	Identify the severity of different behavioral properties and strengthen intervention and training of such behaviors.
(BI, T, L)	Temporal and spatial distribution of unsafe behavior in different behavior individuals.	Identify when and where different individuals are prone to unsafe behavior.

**Table 9:** The process of gas explosion investigation report structure conversion.

Time	Behavioral Trace	Location	Behavioral Property	Behavioral Individual	Degree	Behavioral Action	Specialty
Quarter: fourth	Non-traced unsafe behavior	Blind roadway	Violation of action	Age: 37	Major-risk	Blasting without examining gas concentration	Blasting
Month: November				Working age: 6 years			
Day: 28				Function: grassroots worker, gas inspector			
Shift: ”three and eight” working form, morning shift							
04 11 28 101	2	605	3	103 202 303 0801	2	101	7
Quarter: third	Non-traced unsafe behavior	Tunneling working site roadway	Violation of operation	Age: 32	Major-risk	Turning the local fan on and off casually	Heading
Month: September				Working age: 4 years			
Day: 13				Function: grassroots worker, mine ventilator			
Shift: “four and six” working form, afternoon shift							
03 09 13 202	2	101	2	102 201 303 0702	2	301	1
Quarter: first	Traced unsafe behavior	District return airway	Violation of operation	Age: 41	Medium-risk	Live working/repairing and maintaining energized equipment	Electric
Month: February				Working age: 14 years			
Day: 14				Function: grassroots worker, electrical repairman			
Shift: “three and eight” working form, maintenance shift							
01 02 14 103	1	502	2	104 203 303 0903	3	102	3

**Table 10:** Description and size of clusters.

Clusters	Description	RHS	Count
1	High-risk, traced unsafe behavior in the vicinity of a working face	385	44.20
2	Unsafe behavior related to safety inspection occurring in airway and main haulage roadway	212	24.34
3	Unsafe behavior caused by low-age workers in various rooms	168	19.29
4	Unsafe behavior under medium-risk caused by violations of commanding by managers	106	12.17

**Table 11:** Association rule mining results for the gas explosion accident investigation report.

Rule	LHS	RHS	Support/%	Confidence/%	Lift
Rule1	November	Turning the auxiliary ventilating fan on and off casually	9.76	23.92	1.87
Rule2	Traced behavior	Not evacuating in the case of reduced or stopped air	10.25	35.67	1.76
Rule3	Coal face	Not examining gas concentration in shift time	10.37	43.71	2.70
Rule4	Gas inspector	Blasting without examining gas concentration	11.32	52.34	2.15
Rule5	Major-risk	Blasting without examining gas concentration	11.76	26.86	1.89
Rule6	Heading	Unqualified hole-sealing	9.83	44.73	1.56

Note: the name of equipment or installation contained in unsafe behavior is standard, for they were derived from the current Terms relating to coal mining (GB/T15663-2008), National Standard of the People’s Republic of China.
